# Valedictory Address Delivered before the Graduates of Pennsylvania Medical College: Session of 1842-’3

**Published:** 1843-03-18

**Authors:** 


### BIBLIOGRAPHICAL NOTICE.

*Valedictory Address delivered before the Graduates of Pennsylvania Medical College; Session of* 1842–3. By ROBERT M. BIRD, M. D., Professor of Materia Medica, and Institutes of Medicine. Philadelphia: 1843. 8vo. pp. 12.

This is a short, well written address; presenting, however, we think, too disheartening a picture of the actual condition of the physician in this country. Prof. BIRD thinks medicine has only "reached the point in which it is allowed to *be respectable*." Might he not have added *honourable?* That it is not profitable we fully agree with him, and that the general influence of the profession is not such as the amount of intelligence and moral worth it contains entitle it to, must, we fear, be acknowledged. This Dr. Bird attributes to the standard of scientific attainment not being sufficiently elevated; and the evil, he thinks, is due to the faulty system of medical education pursued throughout the country. No preliminary examination being required of those who are about entering on the study of medicine, there is consequently no evidence of qualification for the severe course of study to be commenced. The shortness of the required term of study is another drawback,—the period being "too short for any but very active, diligent, and well disciplined minds, to travel through the wide circle of the Medical Sciences." The private and hasty method of examination Dr. B. conceives is also disadvantageous.

"The general evil of this system is, its making access to the profession so easy as to seduce many persons to enter, or attempt to enter it, who are destitute of all the requisites essential to the formation of a true medical character; and the profession is insulted by the prevailing idea that any body may be made a physician. This is a very fatal error; but it drives to the threshold of medicine many incompetent persons,—incompetent from want of education and want of talent,—among whom it would be wonderful if some were not permitted to enter. It drives many, even, who have not complied with the moderate requisitions of the established system. The American colleges call only for three years of study; a term which students and their friends sometimes presume to consider superfluously long. They require that this period of study should be passed in the office of a respectable physician,—not merely that the student may be directed in his reading by an instructor, but that he may be carried to the bedside of the sick, in order to be made practically acquainted with disease, and with the duties which he will soon be called on to perform: and yet it has happened that students have even regarded this requisition as useless, and presented themselves as candidates for graduation, without complying with it—as if books and lectures *alone* could make them physicians,—as if this requisition, always considered a most important one, were made only in jest, and never meant to be enforced. In fact, medicine seems to be regarded as a very good sort of business, easily entered, and perfectly well adapted to those who are not well qualified for any other.

"The admission of such incompetent persons, sometimes effected by accident, by error, by false humanity,—perhaps, in some quarters, by a want of regard for the solemn obligations incurred by those who grant medical degrees—is productive of various ill consequences by which the community suffers, and the profession is depreciated. It is to the introduction of individuals not of the highest tone of manners and feeling, that we owe so many of the petty quarrels, the mean jealousies, the tricks of rivalry and hostility, for which the profession is ridiculed ; while baser spirits, imitating the examples of successful quackery, by which we are continually surrounded and *they* seduced, appear, by their own degradation, to sink the whole profession down to the level of their wretched rivals."

Another cause which has apparently escaped our author's attention, and whose influence is by no means insignificant, is individual ambition, and its necessary consequence, the multiplication of schools and teachers, with inordinate and destructive competition; thus flinging open the portals of the profession to all who choose to enter—quantity, not quality being looked to. In all the medical schools within the sphere of our immediate observation there has been a steady progressive improvement in the system of medical education, within the past few years. The facilities and opportunities for instruction are much greater than they were even three or four years since, and there is a general disposition among students—at least those visiting our city—to avail themselves fully of them.

We trust the gloomy anticipations of our author, which we confess we cannot share, will not be realized; that our profession will continue to be *respectable;* and that its high claims will be practically as well as abstractedly confessed, at least in this country.

